# Mapping stakeholders, services, data, and the information system for adolescent health in the West Bank

**DOI:** 10.1186/s12978-025-01991-6

**Published:** 2025-05-31

**Authors:** Aisha Shalash, Maysaa Nemer, Niveen Abu-Rmeileh, Mohammad Kittaneh, Dervla Kelly, Khalifa Elmusharaf

**Affiliations:** 1 Institute of Community and Public Health, Birzeit University, Birzeit, Palestine; 2 School of Medicine, University of Limerick, Limerick, Ireland; 3 Department of Public Health, College of Health Sciences, Qatar University, Doha, Qatar; 4 Department of Geography, Birzeit University, Birzeit, Palestine; 5 Public Health Program, University of Birmingham, Dubai, United Arab Emirates

**Keywords:** Adolescent health, Occupied Palestinian territory, West Bank, Health information system

## Abstract

**Background:**

Adolescent health plays a crucial role in shaping lifelong well-being, yet significant gaps exist in addressing adolescent health needs. In conflict-affected regions like the West Bank, fragmented service delivery, inconsistent data collection, and lack of coordination between providers undermine the effectiveness of health services. An efficient health information system ensures accurate data collection, stakeholder integration, and evidence-based decision-making. This study aims to map the landscape of available adolescent health services in the West Bank, clarify the key service providers, determine the existing data sources, and describe the health information system supporting adolescent health.

**Methods:**

This study utilized a comprehensive landscape analysis to assess adolescent health services in the West Bank. Stakeholder mapping and interviews identified key stakeholders and assessed their roles in service delivery and the health information system. Ethical approval was obtained, and all participants provided informed consent. Data were collected from different healthcare organizations, including governmental bodies, non-governmental organizations, and private facilities. Thematic analysis was performed on interview data, and geospatial analysis was used to visualize the distribution of services and providers across 11 governorates using ArcMap 10.5.

**Results:**

Governmental bodies, non-governmental organizations, and private entities were the predominant providers of adolescent health services in the West Bank. These services were primarily delivered through healthcare facilities, educational institutions, youth centers, and select population-based programs, including vaccination initiatives and the 121 hotline, which provides free psychological support to victims of violence. The adolescent health information system in the West Bank was fragmented, with inconsistent data collection across providers. Governmental, NGO, and private sector organizations use different data systems. Each type of provider used population-based surveys as the primary source of health data. However, there were limitations in the availability of routine data.

**Conclusions:**

This study represents the first comprehensive mapping of adolescent stakeholders and services in the West Bank. Identifying the existing services accessible to adolescents and their providers establishes a foundation for developing target policies and programs that address the current gap and needs of adolescents in the West Bank.

**Supplementary Information:**

The online version contains supplementary material available at 10.1186/s12978-025-01991-6.

## Plain language summary

In the occupied Palestinian territory, characterized by conflict, a surge of humanitarian organizations has put pressure on the existing strained health system, leading to deficiencies in data consistency and coordination. With nearly 50% of the population under 18, understanding adolescent health needs and tailoring services become imperative. This study aims to map the landscape of adolescent health in the West Bank, exploring provider types, geographic distribution and services offered, and the health information system. Results show that governmental providers predominantly focus on adolescent health services, such as reproductive and mental health services. Youth centers and schools play pivotal roles in health promotion. This research underscores the need for robust health information systems to address adolescent health needs effectively and to provide more coordinated information for measurement and planning purposes. Surveys, particularly population-based ones, are the primary source of adolescent health data. International priorities heavily influence funding decisions, while national priorities have emerged, exemplified by the creation of the Palestinian Adolescent Health Coalition and the Department of School and Adolescent and Youth Health. Challenges include data integration and a need for updated indicators. The study highlights the importance of a comprehensive health information system for effective adolescent healthcare delivery, particularly in humanitarian contexts.

**Supplementary Information:**

The online version contains supplementary material available at 10.1186/s12978-025-01991-6.

## Résumé

**Contexte:**

La santé des adolescents joue un rôle crucial dans la définition du bien-être tout au long de la vie, mais il existe des lacunes importantes pour répondre aux besoins des adolescents en matière de santé. Dans les régions touchées par les conflits comme la Cisjordanie, la prestation de services fragmentée, la collecte de données incohérente et le manque de coordination entre les prestataires compromettent l’efficacité des services de santé. Un système d’information sur la santé efficace garantit une collecte de données précise, l’intégration des parties prenantes et une prise de décision fondée sur des données probantes. Cette étude vise à cartographier le paysage des services de santé offerts aux adolescents en Cisjordanie, à clarifier les principaux prestataires de services, à déterminer les sources de données existantes et à décrire le système d’information sanitaire soutenant la santé des adolescents.

**Méthodes:**

Cette étude a utilisé une analyse globale du paysage pour évaluer les services de santé offerts aux adolescents en Cisjordanie. La cartographie des parties prenantes et les entretiens ont permis d’identifier les principales parties prenantes et d’évaluer leurs rôles dans la prestation de services et le système d’information sanitaire. L’approbation déontologique a été obtenue et tous les participants ont donné leur consentement éclairé. Les données ont été collectées auprès de différents organismes de soins de santé, notamment des organismes gouvernementaux, des organisations non gouvernementales et des établissements privés. Une analyse thématique a été réalisée sur les données des entretiens et une analyse géospatiale a été utilisée pour visualiser la répartition des service set des prestataires dans 11 gouvernorats à l’aide d’ArcMap 10.5.

**Résultats:**

Les organismes gouvernementaux, les organisations non gouvernementales et les établissements privés étaient les principaux prestataires de services de santé offerts aux adolescents en Cisjordanie. Ces services étaient principalement fournis par l’entremise d’établissements de soins de santé, d’établissements d’enseignement, de centres pour jeunes et de certains programmes axés sur la population, notamment des initiatives de vaccination et la ligne d’assistance téléphonique 121, qui offre un soutien psychologique gratuit aux victimes de violence. Le système d’information sur la santé des adolescents en Cisjordanie était fragmenté, avec une collecte de données incohérente entre les différents prestataires. Les organismes gouvernementaux, les organisations non gouvernementales et le secteur privé utilisent des systèmes de données différents. Chaque type de prestataire utilisait des enquêtes basées sur la population comme principale source de données sur la santé. Toutefois, la disponibilité des données courantes était limitée.

**Conclusions:**

Cette étude représente la première cartographie complète des parties prenantes et des services destinés aux adolescents en Cisjordanie. L’identification des services existants accessibles aux adolescents et à leurs prestataires jette les bases de l’élaboration de politiques et de programmes ciblés qui comblent les lacunes et répondent aux besoins actuels des adolescents en Cisjordanie.

## Background

The World Health Organization defines adolescence as the age of 10–19 when they face biological, psychological, and physical changes that create the imprint of a person who will eventually lead an independent adult life [1, 2]. Adolescents must have adequate access to proper comprehensive health services during this rapidly changing, vulnerable developmental stage. Investing in the health of adolescents is like investing in generations to come [1, 3–5]. The WHO defines health services as “any service (not limited to medical or clinical services) aimed at contributing to improved health or the diagnosis, treatment, and rehabilitation of individuals and populations” [6]. Successful health services occur when adolescent experiences and cooperation are integrated into the design process [7]. This ensures all needs and wants are met. While providing comprehensive health services is vital to the upbringing of adolescents and the overall improvement of society, the primary objective should be to guarantee these services are accessible, affordable, confidential, and of high quality [8].

Making proper health services available to adolescents globally could result in health equity for all [9]. Moreover, access to proper health services in low- and middle-income countries (LMICs) is crucial because adolescents in LMICs make up 35–50% of the population. Researchers have suggested that modifying existing interventions to serve as a preventive measure would help provide better health services for adolescents in LMICs [10]. One way that has been found to improve health services is by improving the health information system (HIS) [11]. An HIS is a crucial element of a comprehensive health system that collects, processes, and reports data to support policy-making, program development, interventions, and research [12]. The HIS is considered one of the six building blocks of health systems [13]. A well-functioning HIS can provide policymakers with valuable information to guide evidence-based decisions on the different policies and programs that will aid in improving adolescent health outcomes [14]. An effective HIS enables organizations to efficiently coordinate, monitor, and evaluate the humanitarian response in a timely manner [15].

During humanitarian crises, whether they are human-made or caused by natural disasters, women and children are at a heightened risk of violence and vulnerability. This is worsened by limited access to health care, food insecurity, and an absence of autonomy [16]. Many are required to flee their homes, often seeking shelter in schools and hospitals [17]. Countries’ health systems see a strain, an influx of injuries, deaths, disabilities, and a lack of essential medications [18]. Amidst a rise in global crises, with 12 out of the 22 Eastern Mediterranean region countries dealing with armed conflict, the occupied Palestinian territory—encompassing the West Bank, East Jerusalem, and the Gaza Strip—stands as one of these conflict-affected areas [19].

Approximately 50% of the population (5,350,000) in the West Bank and the Gaza Strip is under the age of 18, with 20% classified as adolescents [20]. This demographic composition characterizes the region as having a predominantly young population and requiring tailored healthcare services [21]. Healthcare delivery is provided by four major healthcare providers in the West Bank: The Palestinian Ministry of Health (PMoH), the United Nations Relief and Works Agency (UNRWA), Non-governmental organizations (NGOs), and the private sector. The PMoH and UNRWA adolescent and youth-friendly health services are integrated into primary health care centers and family-friendly services [22]. The PMoH is the main health service provider. Although there are laws that require private facilities to supply data about their services, there is no process in place to ensure this data is reliable and accurate. The combination of a strained health system in the West Bank with the proliferation of different humanitarian organizations with their data systems has resulted in deficiencies in maintaining consistent data and coordination, delivery, and assessment of the activities undertaken by these organizations [23].

The study originated from the imperative for a robust and efficient HIS that is essential for formulating effective policies to address the health requirements of adolescents in the West Bank. Despite the critical role of adolescent health in shaping lifelong well-being, there is a significant lack of understanding regarding the specific services available, the key stakeholders involved, and how these services are prioritized, particularly in conflict-affected settings like the West Bank. The absence of a coordinated HIS leads to fragmented service delivery, inconsistent data integration, and ineffective health interventions for adolescents. Reinforcing HISs enhances decision-making grounded in reliable data, thereby improving intervention strategies and health outcomes. Understanding the adolescent health services system is crucial for grasping the complexities of the HIS. A previous mapping of sexual and reproductive health services for adolescents in the occupied Palestinian territory highlighted the gaps but did not encompass the full spectrum of services and stakeholders involved [24].

The current research maps the landscape of adolescent health services, identifies key stakeholders, and evaluates the existing adolescent health information system (HIS) in the West Bank. The primary objective is to determine the HIS needs crucial for improving adolescent health, with a focus on enhancing adolescent health outcomes and stakeholder coordination. Additional objectives include providing a comprehensive overview of available services, assessing the role of various stakeholders, and analyzing how current data sources influence service delivery and priority setting.

By mapping adolescent health services, stakeholders, and the HIS, this study aims to clarify how these elements can enhance the coordination, delivery, and effectiveness of the HIS, particularly in humanitarian settings. The findings will inform the development of a comprehensive HIS that addresses adolescent needs, improves health outcomes, and facilitates coordinated healthcare delivery in the West Bank.

## Methods

In order to achieve this, our study adopted a comprehensive landscape analysis methodology to gain an understanding of adolescent health services, with a specific focus on humanitarian settings [25]. The research approach encompassed stakeholder mapping and analysis, as well as key informant interviews, to explain the landscape for health services provided to adolescents. This landscape also includes the role the health information system plays in providing these services in all parts of the West Bank, including urban, rural, and camp settings.

### Stakeholder mapping and analysis

Our study targets stakeholders, including service providers, policy and program decision-makers within various healthcare organizations, governmental bodies, NGOs, and private facilities. In this paper, the analysis of service providers, as one type of stakeholder, is drawn out separately in the analysis section. They refer specifically to stakeholders that directly deliver services to adolescents, such as in clinics, hospitals, youth centers, and schools.

The initial phase of our methodology involved extensive stakeholder mapping and analysis. Using the snowball method, we started with the largest organizations, and then they were able to identify other organizations. We identified and categorized key stakeholders involved in adolescent health services, considering the diverse range of contributors such as healthcare providers (doctors, nurses, mental health professionals), policymakers, educators, community leaders, and individuals from NGOs actively engaged in adolescent health. An adolescent health service provider was determined to be any organization working towards promoting health and minimizing harm to 10–19-year-old individuals [26]. Using a snowball process and conducting internet searches, we mapped stakeholder details. The principal researchers (AS) and (MN) collected the data. Both are fluent in Arabic and have previous experience in conducting stakeholder mappings and landscape analyses. We collected contact information, services and activities offered, indicators, and if any precautions were taken to maintain the confidentiality of patients and participants. All details of each stakeholder found on the internet were then verified by phone or in person by the researchers. The stakeholder details sheet used as a guide for the collection of information can be found in Additional file 1. Stakeholder mapping and analysis served as the first step to identify each stakeholder’s role as either a leading, collaborating or contributing, or funder within the adolescent health services landscape. This mapping and analysis provided a foundation for further recognition of the services that should be accommodating to adolescent health needs.

### Key informant interviews

Building upon the insights derived from stakeholder mapping, we conducted key informant interviews and gained invaluable information from stakeholders. AS and a trained research assistant conducted the interviews. Both had prior experience in qualitative data collection and are also fluent in Arabic, which enabled effective communication with the participants. These insights enabled a comprehensive analysis of providers and services, thereby enriching the depth and strengthening the accuracy of our findings. The key informants included representatives of the identified organizations from diverse backgrounds, including healthcare professionals, policymakers, educators, and NGOs, offering a view of the adolescent health services landscape.

Data collected through these interviews captured in-depth perspectives, experiences, and expert opinions about the adolescent health services offered and the role of the health information system in the design and delivery of these services. The interviews were semi-structured, allowing for the questions to be adapted to discuss how the organizations selected services and what kind of health information system is employed. Interviews were done until saturation was reached—no new information from stakeholders was retrieved.

### Data analysis

The stakeholder mapping information collected was extracted using Excel. The data collected through key informant interviews underwent thematic analysis using MAXQDA [27]. The interviews were transcribed, and codes were developed by two researchers (AS, KE). The findings were systematically organized to draw meaningful conclusions regarding the current state of adolescent health services and the health information system involving adolescent health. Themes were organized as service availability, data sources, challenges in the health information system, and gaps in service delivery. To validate the findings, we triangulated the data from key informant interviews with stakeholder mapping and secondary data sources, ensuring the accuracy and reliability of the conclusions. This process allowed us to cross-check different sources of information and build a more comprehensive understanding of the services provided and gaps in the system. The methodology for conducting the key informant interviews was the same as that described in our earlier publication [28], where we detailed the interview process, participant selection, and analysis.

### Geovisualization method

To geovisualize the findings from the stakeholders’ and providers’ mapping and analysis, we used the geographic information system (GIS) software ArcMap 10.5. This visualization aimed to show the distribution of adolescent services and providers among the 11 Governorates in the West Bank. It will help understand where services are mostly concentrated and whether certain services are scarce in some areas of the West Bank.

To achieve this geospatial visualization, we needed to add three main data sources to the ArcMap software. First, data from the Palestinian Central Bureau of Statistics (PCBS), a reputable organization known for providing a wide range of statistical data for the occupied Palestinian territory, was taken. The data used in this study covers the period up to the year 2022, ensuring the inclusion of the most recent and up-to-date information about the organization and providers within the West Bank and their distribution in the different Governorates, which was available during the analysis phase. Prior to analysis, the data obtained from PCBS underwent preprocessing and preparation to make it compatible with ArcMap 10.5. Second, to obtain the necessary data layers (shapefiles) at the governorate level, the official website for the spatial data of the Ministry of Local Government (MoLG), called GeoMoLG, was accessed. Third, data collected about the number of services and providers were classified by Governorates based on the information obtained about each service. This classification was done using Excel sheets, which were organized in the same order as the other two components (PCBS data files and the shapefiles) so that for each of the 11 Governorates of the West Bank, the number of providers (for each of the four main providers) and the number of services (for each of the five main services) were identified.

To effectively communicate the desired information, careful consideration was given to the map design and symbolism used for the thematic maps. These design choices considered both the intended audience and the research objectives. By utilizing ArcMap 10.5, the collected quantitative data was visualized and analyzed in a spatial context. This approach facilitated the creation of meaningful visual representations that supported the research objectives and provided valuable insights.

### Ethical considerations

This research obtained ethical approval from the Ethical Research Committee of the Institute of Community and Public Health at Birzeit University, with reference number 2019 (4–2). Prior to their involvement, each participant offered verbal consent, acknowledging that they retained the right to terminate the interview at any stage. The recording of interviews started after participant consent was given, and individuals were able to stop the recording at any point they felt was necessary. Participants were guaranteed that their interviews would be treated with the utmost confidentiality, ensuring the preservation of their anonymity.

## Results

Twenty-one key informant interviews were conducted with different stakeholders engaged in the HIS related to adolescent health. Table 1 provides the characteristics of the stakeholders interviewed.

**Table 1:** Characteristics of stakeholders: interview length and stakeholder category

Identification (ID) number	Interview length (min)	Stakeholder category	Sex of stakeholder	Years of experience
1	81	Governmental	Male	28 years
2	42	Local NGO	Female	12 years
3	27	Local NGO	Female	14 years
4	35	Local NGO	Male	33 years
5	39	International NGO	Female	20 years
6	58	Local NGO	Male	14 years
7	47	Governmental	Female	15 years
8	22	Governmental	Male	22 years
9	30	Governmental	Female	17 years
10	48	Governmental	Female	14 years
11	38	Governmental	Female	3 years
12	29	Governmental	Male	5 years
13	33	Governmental	Female	27 years
14	29	Governmental	Female	8 years
15	23	International NGO	Male	13 years
16	94	International NGO	Female	20 years
17	55	International NGO	Female	5 years
18	26	International NGO	Female	15 years
19	32	Local NGO	Male	12 years
20	49	Local NGO	Male	23 years
21	43	Local NGO	Male	24 years

### Service availability

#### Stakeholder mapping

Over 300 different organizations’ websites, social media pages, and contact information were explored during the stakeholder mapping. The stakeholders identified were categorized into four main types: governmental, private organizations, NGOs, and municipalities. It was found that 35 major organizations provide adolescent health services in the West Bank.

**Fig. 1:**
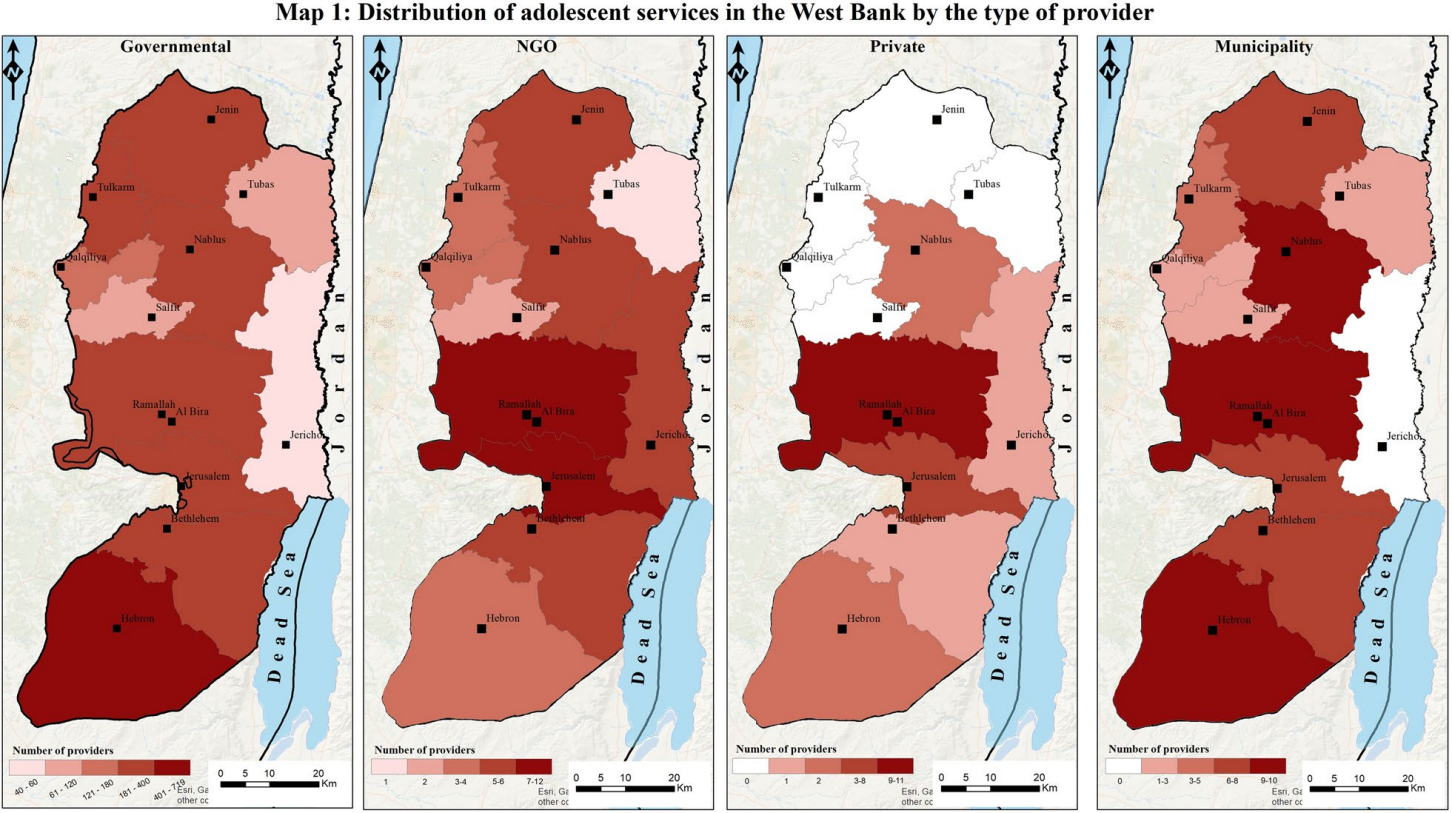
Distribution of adolescent services in the West Bank by the type of provider

**Fig. 2:**
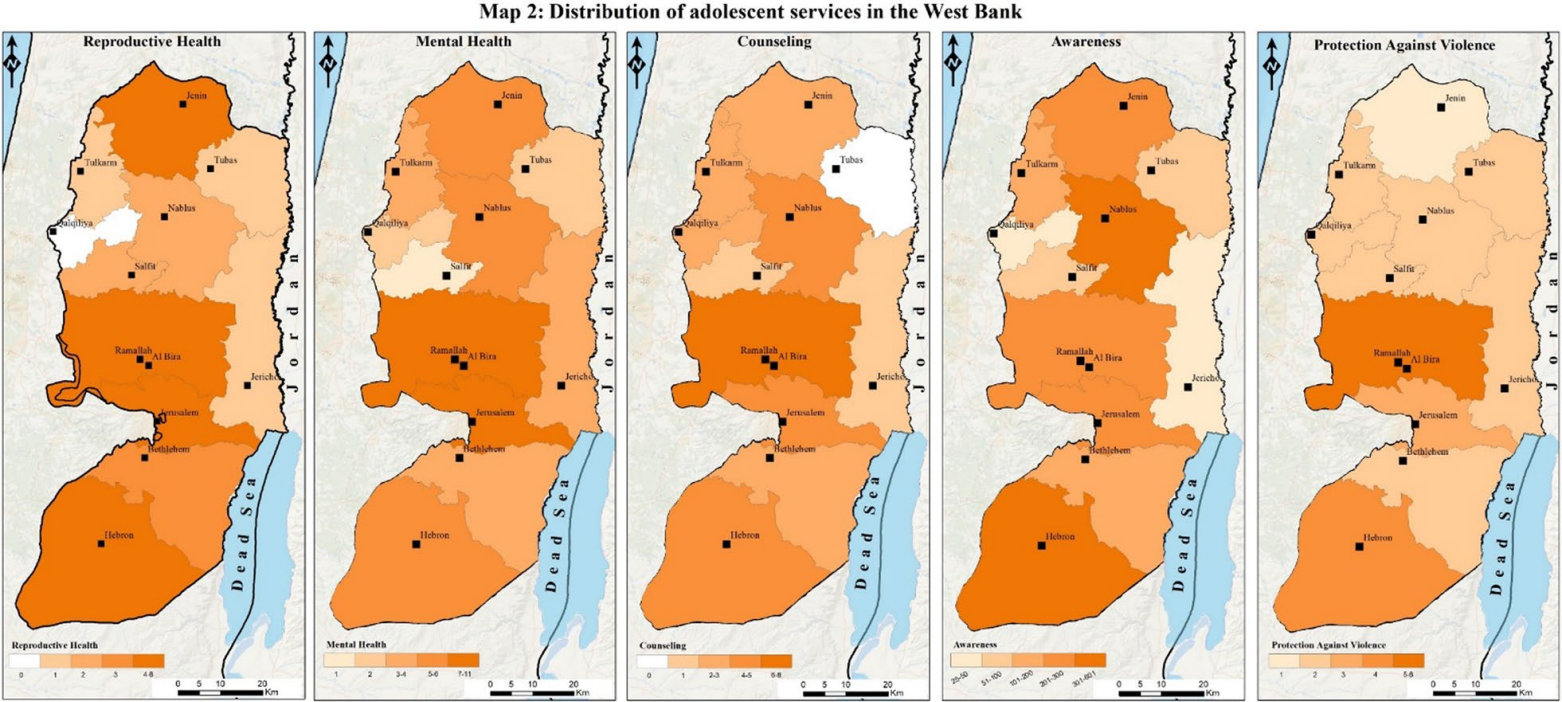
Distribution of adolescent services in the West Bank

Governmental organizations, making up most of the stakeholders (94.7%), are responsible for providing essential health services, often through health facilities and schools. Private organizations (0.85%) offer limited but specialized services, often in collaboration with larger agencies. NGOs (2.05%) are actively engaged in awareness campaigns and targeted interventions for adolescents. Municipalities (services in towns or cities) (2.3%) provide services through youth centers and population-based initiatives. The maps in Fig. 1 illustrate the distribution of these providers throughout the West Bank. These services usually occur in health facilities, schools, and youth centers or are population-based services. The major funders were found to be the United Nations Population Fund (UNFPA) and the United Nations Children’s Fund (UNICEF), while organizations like Oxfam and Save the Children are newly focusing on adolescent health needs. Municipality-run youth centers play a huge role in providing health awareness, counseling, and vocational training. Table 2 shows a breakdown of the major organizations that provide different adolescent health services in the West Bank and their characteristics. The five most mentioned services provided, with their respective overall percentages in the West Bank, were awareness (93.9%), mental health (2.2%), reproductive health (1.3%), counseling (1.3%), and protection against violence (1.1%). The percentages represent the proportion of service delivery points providing said services. Awareness services are health education on various topics, such as reproductive health and nutrition, and they are given in groups, usually in schools or summer camps. Mental health services refer to individual counseling or psychological support. Reproductive health services also refer to individual medical services that are given to boys and girls, usually in a clinical setting, such as family planning. We were able to map these as well, and the distribution within the West Bank can be found in Fig. 2**.**

**Table 2:** Major organizations providing adolescent health services in the West Bank and their characteristics

Name of stakeholder-organization	Type of organization	Location of services	Health services offered*						
			Sexual and reproductive health	Mental health	Counseling	Protection against violence	Awareness (educational activities)	Nutrition	Vocational and social rehabilitation
1. Ministry of Health	Governmental	Schools, Health Facilities, Population Based Services	L	L	L	L	L	L	
2. Ministry of Education	Governmental	Schools, Population Based Services		C	L	C	L	L	L
3. Ministry of Social Development	Governmental	Schools, Health Facilities, Population Based Services, Youth Centers		C	C	L	C		L
4. Higher Council for Youth and Sports	Governmental	Schools, Youth Centers			L		L		L
5. UNRWA	NGO	Schools, Health Facilities	L	L	L	L	L	L	L
6. SAWA	NGO	Schools, Health Facilities, Population-based Services	C	C	C	C	C		C
7. UNFPA	NGO		F	F	F	F	F	F	
8. UNICEF	NGO		F	F	F	F	F	F	
9. Y-PEER Youth Peer Education Network	NGO	Schools, Health Facilities, Youth Centers	C	C	C		C		
10. Good Shepherd Youth Club Jericho (Nadi Shabibet Al-Rai Al-Saleh-Ariha)	NGO	Youth Centers		C	C		C		C
11. Palestinian Medical Relief Society	NGO	Schools, Health Facilities, Youth Center	C	C	C	C	C	C	C
12. Union of Health Care Committees (UHCC)	NGO	Health Facilities, Youth Centers	C	C	C	C	C		
13. Juzoor for Health and Social Development	NGO	Schools, Health Facilities, Youth Center		C	C	C	C	C	C
14. Hebron Youth Development Resource Center (YDRC)	NGO	Schools, Health Facilities, Youth Centers	C		C		C		C
15. Youth Development Association (YDA)	NGO	Youth Centers			C				C
16. Young Men’s Christian Association (YMCA)	NGO	Schools, Health Facilities, Youth Centers	C	C	C	C	C		C
17. Independence Youth Union	NGO	Youth Centers					C		C
18. Al-Ofoq foundation for youth development	NGO	Youth Centers					C		C
19. Al-Sadiq Al-Taieb Association	NGO	Schools, Health Facilities, Youth Centers		C	C		C		C
20. Caritas Center	NGO	Schools, Health Facilities, Youth Centers	C	C	C		C		
21. Save the Children	NGO	Schools, Health Facilties, Youth Centers	C	C	C	C	C	C	C
22. Oxfam	NGO		F	F	F	F	F		
23. Sharek	NGO	Schools, Youth Centers			C		C		C
24. Palestinian Family Planning & Protection Association (PFPPA)	NGO	Health Facilities	C	C	C	C	C		
25. Palestinian Counseling Center	NGO	Health Facilities		C	C	C	C		
26. Sourif Municipality	Municipality	Youth Centers			C		C		
27. Ramallah Municipality	Municipality	Schools, Youth Centers			C		C		C
28. Al-Bireh Municipality	Municipality	Schools, Youth Centers			C		C		C
29. Taqoua’ Municipality	Municipality	Schools, Youth Centers			C		C		
30. As-Samua' Municipality	Municipality	Schools, Youth Centers			C		C		
31. Kufr Thulth Municipality	Municipality	Schools, Youth Centers			C		C		C
32. Kufur Rai' Municipality	Municipality	Schools, Youth Centers			C		C		
33. As-Sawahreh Ash-Sharqiyeh Local Council	Municipality	Schools, Youth Centers			C		C		
34. Beit Ula Municipality	Municipality	Schools, Youth Centers					C		
35. Tomorrow’s Youth Organization	NGO	Youth Centers	C	C			C		C

^*^L = Leading services, F = Funding services, C = Collaborating

The analysis of the distribution of adolescent health services across the West Bank, as illustrated in Figs. 1 and 2, reveals several patterns. Services are predominantly concentrated in certain areas, reflecting the centralization of resources and infrastructure in these regions. This bias is evident in the clustering of facilities and programs in cities and towns, leaving rural regions underserved. The unequal geographic distribution leaves adolescents in certain areas facing limited access to essential health services. The type of service provider also shows patterns in governmental organizations providing most of the adolescent health services. NGOs, while fewer in number, appear to target areas with identified gaps in governmental service delivery, suggesting a complementary role. Private organizations and municipality-run youth centers provide additional, limited services focused on specific health needs or community initiatives. The presence of multiple providers in the same region, however, raises concerns about potential overlap and duplication, highlighting the need for improved coordination among stakeholders.

The maps demonstrate that specialized services, such as mental health and reproductive health, are concentrated in areas, while rural areas are primarily limited to general health awareness and counseling. These patterns collectively point to the urgent need for targeted interventions to expand adolescent health services in underserved regions and strengthen coordination mechanisms to optimize resource use and service delivery. After a comprehensive understanding of the major organizations and their distribution, the following exploration focuses on the specific settings where adolescent health services are delivered: health facilities, schools, youth centers, and population-based services.

### Service settings

#### Health facilities

Adolescent health services are provided in all primary health care clinics and mobile clinics as part of family health services and not exclusively for adolescents. Primary health care services are mainly offered by the PMoH, UNRWA, the Palestinian Medical Relief Society (PMRS), and the Union of Health Care Committees (UHCC). The PMRS mobile clinics are also available to provide medical and psychological consultations to underserved communities and regions with inadequate medical care. These clinics are staffed by a general practitioner who performs medical examinations and consultations and a psychologist who provides psychological consultations. The locations of these clinics are determined according to an annual plan, ensuring that they are accessible to those who require them the most.

#### Schools

As of the 2022/2023 school year, there are a total of 2394 schools in the West Bank. These include 1896 governmental, 96 operated by UNRWA, and 402 private schools. In the school-focused health initiatives, the organizations prioritize comprehensive health education for adolescents, directing efforts towards 9th to 11th-grade students in all government and private schools on an annual basis. The seamlessly integrated school health program is an integral part of their broader child health initiative, offering health checks for students up to the age of 18. They encompass students from kindergarten to 12th grade, addressing critical issues such as adolescence and early marriage awareness. Recognizing the pivotal role of nutrition in schools, policies underscore balanced nutrition and the promotion of healthy habits. Implementation of health awareness campaigns, electronic monitoring systems for students' nutritional status, and vaccination programs for 6th, 8th, and 9th-grade students all align with the overarching commitment to comprehensive adolescent health within the school environment.

#### Youth centers

Youth centers in the West Bank, operated by community-based organizations, serve as invaluable hubs providing free services for adolescents and youth, fostering a secure environment for skill development and leisure activities. We were able to map ten youth centers throughout the West Bank, and they were mainly municipality initiatives. These centers deliver educational programs spanning gender equality, mental health, and sexual/reproductive health, with a particular emphasis on encouraging peer-driven knowledge exchange. Complementing these initiatives are awareness programs addressing various health topics. Specific programs, such as the Youth Empowerment Program for girls aged 14–18 and the Big Brother-Big Sister Program, offer targeted support, aiding adolescents in overcoming challenges like social stigma and gender-based violence. These centers not only strengthen community and familial bonds but also provide psychological assistance for issues ranging from sexual/reproductive health to family relationships, ultimately empowering adolescents to navigate societal pressures authentically.

In response to the evolving healthcare landscape in the West Bank, essential services have been established that are accessible to all residents, irrespective of their affiliation with health facilities, schools, or youth centers. Among these newly introduced services is a comprehensive vaccination program, which extends free vaccination services to all children and adolescents. In addition to the introduction of the 121 Hotline by the NGO, Sawa addresses medical concerns by offering free consultations for individuals of all ages, with a dedicated focus on child protection for those under 18. This listening line, complemented by a user-friendly phone application, facilitates virtual appointments with healthcare professionals, which are bookable through WhatsApp. Notably, the 121 Hotline has forged partnerships with major social media platforms, including Facebook, Instagram, and TikTok, enabling the swift removal of harmful content, electronic bullying, or inappropriate material within 72 h.

### Gaps in service delivery

Significant gaps exist in the delivery of services in the West Bank, such as comprehensive sexual education and contraceptive access. Comprehensive sexual education programs are absent, leaving adolescents without a full understanding of sexual health. In primary healthcare clinics, married adolescent girls have access to contraceptive services, but these are not available to unmarried adolescents. Data collection in health facilities is limited because adolescents, who are generally healthy, rarely attend these facilities for services. Health information related to awareness and counseling is typically not recorded, further limiting the availability of comprehensive data on adolescent health.

Many services for adolescents are funded based on international priorities rather than local needs. These services often depend on project-based funding aligned with donor priorities, which may not necessarily reflect the specific needs of the local population. The reliance on external funding results in a lack of continuity when priorities shift and was highlighted by funders. When asked how they decide on the projects to implement, the majority indicated that the availability of external funding primarily drives their decisions.

“We mainly fund projects, but we do not provide direct services. Our funds usually come from a call or grant that we have applied for and are granted. We then find the most appropriate organization to provide this service.” Stakeholder ID # 16.

### Data sources

#### Population-based surveys/census

Most of the adolescent health statistics come from population-based surveys, which are household and school-based. The most popular school-based surveys are the Global Health School Survey and the Global Youth Tobacco Survey. The Multiple Indicator Cluster Survey in occupied Palestinian territory only included married women 15–19 from the adolescent age group. A youth survey was also done in Palestine, but the focus was on the ages 18–29. The last census was in 2010, and no adolescent health statistics were obtained from the survey. Surveys are the most used source to understand behavior risk facts such as smoking, nutritional status, educational level, and employment. Population-based surveys are funded by organizations such as UNICEF and UNFPA.

“An organization comes to the PCBS and asks us to do this survey…This is how we decide which surveys are done.” Stakeholder ID #19

A gap of information in the population-based surveys was found amongst the 10–14 age group. When asked about why there was a lack of data and indicators for this age group, a few of the stakeholders recalled that the country's definition of adolescents starts at the age of 15.

“You will find a gap in the health information of 10–14 because the country defines adolescents and youth as starting at the age of 15. You will find information on work indicators even though the age starts at 15 because 10–14 years is considered to be child labor and taken into consideration.” Stakeholder ID #20.

### Challenges in the health information system

A variety of providers offer adolescent health care services. Each provider is tasked with gathering data from their respective facilities, such as the type of service provided, the number of adolescents treated, and supply statistics. This data must be included in the annual report. However, providers utilize different health management information systems, with the Ministry of Health facilities using AVICENNA in hospitals and DHIS2 in primary health care clinics. This variance in systems presents a challenge for integrating data from primary and secondary facilities. When the different organizations were asked about the system of sharing data with others, all answered that they submit an annual report to the MoH every year.

“We submit an annual report to the Ministry of Health every year.” Stakeholder ID #6

“There is a yearly report, and in the yearly report, we give a summary of the statistics and what programs were offered and who attended these programs.” Stakeholder ID #18.

When asked if data is used to decide if a program is a priority, many organizations described using internal needs assessments. When the researchers asked for a copy of these assessments, they were told they were not for publication. It was clear that in most organizations, some monitoring and evaluation is used but has not been published.

“We do need assessments, before and after, to monitor and evaluate our projects internally…. These reports are not published.” Stakeholder ID #4

Also, many health organizations and providers do not have a unified system for data sharing, which results in fragmented data collection efforts, making it hard to make evidence-based decisions. Many stakeholders reported adolescent health data comes from population-based surveys, and the available indicators are derived from these surveys.

In the final phase of the interviews, key informants were queried about the utilization of adolescent health indicators in the decision-making process for implementing services. Several stakeholders expressed concern over the absence of up-to-date adolescent health indicators, emphasizing the necessity of adopting priority indicators. It was noted that adolescent health services, being a new focus, have garnered increasing interest from international organizations and funders in recent years.

“We prioritize which indicators we should focus on, which ones are more relevant and publicize it to all organizations. This will help us when we design new projects, interventions, and programs, refer to these indicators, and see that they are the most relevant to the Palestinian context. Those are the more relevant to SDGs and to the future interventions that we want to make, and then we can unify them for all the organizations.” Stakeholder ID #3.

When asked what indicators they would like to see available on the priority list, a few stakeholders mentioned the importance of mental health indicators as well as employment indicators.

"… Unfortunately, they are out there, and nobody knows what they are doing. So, I think statistics related to adolescence in the labor force would be another important indicator to see how it is influencing their future and how that is influencing their health status, mental status, you know, social, and economic status." Stakeholder ID #15.

"Definitely, mental health and psychosocial status of science would be another area, and indicators around that would be important given the reality. Unfortunately, that and the Palestinian people are facing, you know, violence on a daily basis, and the mixed results most recently has also indicated the increase of domestic violence." Stakeholder ID #13.

The findings of this study offer an understanding of the adolescent health services landscape, highlighting gaps in service delivery and coordination. These results serve as a foundation for discussing the implications of improving adolescent health outcomes and strengthening the HIS in the West Bank for evidence-based service allocations.

## Discussion

The stakeholder mapping involved exploring over 300 organizations and revealed 35 major organizations that delivered adolescent health services in the West Bank. Primary funders included UNFPA and UNICEF, with emerging focus from Oxfam and Save the Children. Youth centers play a significant role in health awareness and vocational training. Reproductive health, mental health, counseling, awareness, and protection against violence were the services that were most provided. Settings for service delivery included health facilities, schools, and youth centers. Key informant interviews revealed international priorities influencing funding decisions, while surveys and health facility data shaped national priorities. Challenges included integrating data from diverse systems and a need for updated indicators.

Understandably, the most provided services were reproductive health, mental health, counseling, and protection against violence, as these are found to be the most needed services for adolescents in humanitarian settings [29–31]. The primary types of services that are offered are awareness programs, which align with most adolescents being healthy and needing programs to advocate prevention and not treatment [32]. Advances in reproductive health in humanitarian settings have improved access to services, but significant gaps remain in maternal and newborn health, family planning, gender-based violence, and HIV/STIs [33]. Water, sanitation, and hygiene (WASH) interventions are also considered to be important [34], but no adolescent WASH interventions were found or documented in the West Bank. A great focus is placed on reproductive health and mental health during crisis times because adolescents are more vulnerable to risks and conditions and sometimes must play the role of an adult before passing through the developmental phase of adolescence [35]. This occurred in Lebanon amongst Syrian refugees, with adolescents dropping out of educational establishments and adolescent girls getting married [36]. The Palestinian Educational System is currently working on incorporating sexual education, mental health, gender roles, and gender equity and equality into the educational curriculum [37]. There currently is no sexual education program in the current system. Furthermore, two studies have been done on the perception of sexual education and knowledge of university students in different geographical regions in the occupied Palestinian territory [38, 39]. A potential reason for the disparity in SRH knowledge was noted between students who attended private schools in the occupied Palestinian territory, where co-educational settings and some sexual education might be provided [38, 39].

Developing programs and encouraging service delivery in these areas is essential. Mobile clinics are used in some rural areas, but one recommendation could be the use of telehealth. In a systematic review done to measure the satisfaction of rural patients with telehealth, it was found that they were satisfied with their access to health care and had less travel time [40]. A hotline is currently available, but it would be necessary to advertise widely as most adolescents do not know about this service. A current evaluation of the hotline's use would allow for suggestions to improve and increase use. Schools and youth centers are found to be the best places for adolescents to receive services, given that is where they spend most of their time. Youth centers can be an alternative to health facilities as most adolescents are seen as healthy and are less likely to go to health facilities, especially girls, in fear of the stigma of being unwell [41].

With the main programs being of international priority, there can sometimes be seen as a lack of local ownership, leading to unsustainability of the programs lasting beyond the funding of the programs being offered [42]. A recent study described the health response in Yemen as being more effective when the local community was involved, allowing for the sustainability of health services in the future [43]. It has also been observed that involving local youths in humanitarian responses and providing them with support within the system elicits a more positive reaction, fostering a sense of belonging during challenging times. This was evident in Sierra Leone during the response to the Ebola outbreak [44]. Integrating locals into the decision-making processes would be essential. It is vital to invest in the response capacity of national NGOs, which allows for a more tailored approach to addressing specific local needs rather than adhering strictly to international agendas [45]. This shift enhances program effectiveness and strengthens local capabilities, ensuring the sustainability and relevance of interventions. By capitalizing on local knowledge and expertise, international donors can better align their strategies with the actual health needs and priorities of adolescents in the West Bank, fostering a more collaborative and impactful approach to health service delivery. Also, it has shown that programs are more effective when local actors are involved in the coordination, execution, and monitoring [43].

During these times, humanitarian organizations require pre-humanitarian data to help plan their response [46]. The situation is seen as a lack of well-defined indicators concerning adolescents, especially in low- and middle-income countries [47]. The Global Action for Measurement of Adolescent (GAMA) health initiative has identified 52 priority adolescent health indicators [48]. Still, it has yet to test the feasibility and reliability of these indicators in humanitarian settings. Some of the included priority indicators are the prevalence of obesity, tobacco use, alcohol use, and anemia. Integrating adolescent health indicators and data more seamlessly into the health information system will facilitate the sharing of information, enabling a more effective response to the specific health needs of adolescents [49]. Currently, population-based surveys are identified as the most effective means of gathering comprehensive data on adolescent health, primarily targeting older adolescents 15–19 years old. However, a lack of data on younger adolescents aged 10–14 years is evident in most low-and-middle income countries, not just in humanitarian settings. Conducting surveys for this younger age group is often challenging due to their classification as children, but it is feasible to collect through school-based surveys [50].

Modifying existing surveys to include younger adolescents and males would help increase data on these age and sex groups. Instead of using household surveys, we should use school surveys to effectively reach this age and sex group, taking into consideration that you will be losing out-of-school adolescents. Using online surveys will help increase anonymity and allow adolescents to answer questions more honestly compared to being in school. It would be essential to be creative in data collection techniques.

## Conclusion

In conclusion, this study underlines the critical importance of developing and integrating a comprehensive health information system to enhance the coordination, delivery, and effectiveness of adolescent healthcare services in the West Bank, particularly within humanitarian contexts. By improving accessibility, coordination among healthcare providers and humanitarian organizations, and the availability of timely data, such a system holds the potential to impact adolescents' health outcomes significantly.

The triangulation of stakeholder mapping and key informant interviews ensured a holistic exploration of the factors influencing the provision of health services to adolescents, contributing valuable knowledge to the existing literature and informing how the health information system can be used to make informed program decisions. The main challenge of the health information system regarding adolescent health was data integration. Needs assessments done by NGOs, being shared, will help combat repetition and will allow for resources to be used for other needed programs. The adoption and development of comprehensive adolescent health indicators to enhance decision-making and improve the monitoring and evaluation of programs is important. GAMA has identified a draft of the priority list, and it is important to determine the current indicators that are currently available and the feasibility of collecting these indicators in the current context. Further research will help determine the strengths and challenges the current health information system has in tailoring to the needs of adolescent health.

### Strengths and weaknesses

One of the main strengths seen was the ability to accumulate major stakeholders of adolescent health in the West Bank and verify the available information—adding main stakeholder input allowed for a comprehensive understanding of the adolescent health service landscape. A more comprehensive study, including the Gaza strip, is needed. Although we are aware of certain stakeholders servicing Gaza, a comprehensive mapping of their involvement was not feasible within the scope of this study.

## Supplementary Information

Supplementary Material 1.

## Data Availability

Data are available from the “Data Access Committee at ICPH-BZU, represented by the ICPH Ethics Review Committee (contact via icph@birzeit.edu) and the corresponding author" for researchers who meet the criteria for access to confidential data. Data cannot be shared publicly because all interviews must be anonymous. The participants, who are government employees and hold other important positions in the country, would only agree to the interview if their identity was kept anonymous.

## Abbreviations

GAMA: Global Action for Measurement of Adolescent
GIS: Geographic Information System
MoLG: Ministry of Local Government
NGOs: Nongovernmental Organizations
PCBS: Palestinian Central Bureau of Statistics
PMoH: Palestinian Ministry of Health
PMRS: Palestinian Medical Relief Society
UHCC: Union of Health Care Committees
UNFPA: United Nations Population Fund
UNRWA: United Nations Relief and Works Agency for Palestine Refugees in the Near East
UNICEF: United Nations Children’s Fund
WASH: Water, Sanitation, and hygiene
WHO: World Health Organization
