# Supplementary material for: Mapping stakeholders, services, data, and the information system for adolescent health in the West Bank
Source: Reprod Health. 2025 May 31;22(Suppl 1):81. doi: 10.1186/s12978-025-01991-6 (PMC12125755; doi:10.1186/s12978-025-01991-6)
Supplement: Supplementary file 1 — Supplementary Material 1. [file 12978_2025_1991_MOESM1_ESM.docx]

Additional File 1: Guide for collection of Stakeholder details

Stakeholders Details

Date:

Name of information source:

Position:

Telephone number:

1. **Stakeholder name**
2. **Stakeholder category**

Q: (Which sector do you belong to?)

(لأي قطاع تنتمون؟)

- Governmental (organizations that are owned and operated by the government and exist to provide services for its citizens)
- Private
  - For-profit/Commercial
  - For- nonprofit non-governmental:
- UN related organizations
- Others.

1. **Scope**

Q: (What is the scope of your work?)

(ما هو نطاق عملكم؟(محلي/وطني/دولي)

- International
- Local
- National

1. **Stakeholder subgroup**

Q: (Which unit you belong to? (If any))

(تحت أي وحدة تندرجون؟(إذا يوجد))

1. **Contact information (headquarters):**
   - Telephone number(رقم الهاتف) :
   - Fax :(الفاكس)
   - Email:
   - Full address :(العنوان بالكامل)
   - Website :(الموقع الإلكتروني)
   - Facebook:
   - Twitter:
   - Working hours/days :(أيام/ ساعات العمل)
   - Responsible person (adolescent-related project manager) :(الشخص المسؤول)
2. **Do you provide health services for ages ranging between 10 to 24 years?**

)19 (سنة هل تقدمون خدمات صحية للفئة العمرية بين10 إلى

**(If yes)(إذا نعم)**

1. **Type of services / age and gender of the target population**

Q: (What are these services and which age group and gender they target? (

(ما هي هذه الخدمات ولأي فئة عمرية وجنس تقدم؟)

1. **Location**

Q: do you have more than one center to deliver your services?

(هل لديكم أكثر من مركز واحد لتوفير خدماتكم؟)

1. **Approaches for service delivery**

Q: (Is the service exclusively offered for adolescents?)

(هل تقدم هذه الخدمات بشكل حصري للهذه الفئة العمرية ؟)

- (If yes )Targeted Approach

The targeted approach refers to a situation where services are designed and planned for adolescents and youth alone and are offered in settings that meet only the needs of the adolescents and youth and do not include other groups. Such services may be clinical, non-clinical, or a combination of both

- (If No)Integrated/Mainstreamed Approach

A situation where adolescents and youth receive services as part of the general public, but special arrangements are made to make the services more acceptable to them and all service providers are sensitized on adapting service delivery to adolescents’ and youth needs as part of their definition of quality care. This mainstreamed approach can be adapted at any level of health facility, including primary health care facilities.

1. **Service delivery points**

Q: (Where you offer the services delivery points?)

(أين توجد نقاط تقديم الخدمة؟)

(ما هو نوع الأماكن التي يتم تقديم الخدمات فيها؟)

(أين يمكن للشباب/ اليافيعين/المراهقين الحصول على هذه الخدمة؟)

1. **Referral elsewhere/coming back**

Q: (Does the service require referral to other service providers or coming back)

(If it is non- governmental: Do you refer to / receive cases from government?)

هل تتطلب هذه الخدمات التحويل إلى مزودين خدمات اخرين أو هل تتطلبهذه الخدمات الرجوع مرة أخرى؟) )

إذا كانت المؤسسة غير حكومية ،: هل تحول / تستقبل التحويلات من الحكومة) )

1. **Fees**

Q: (How much the person has to pay to use this service?)

(كم يجب على الشخص أن يدفع ليستفيد من هذه الخدمة؟)

(ما هو الملبغ المطلوب للدفع للاستفادة من الخدمة؟)

1. **Insurances**

Q: (Is there an insurance which cover this service? If yes specify)

(هل يوجد تأمين يغطي هذه الخدمة؟إذا كانت الإجابة نعم هل يمكن أن توضح ما هي الجهات التي توفر هذا التأمين؟)

(هل تتعاملون مع شركات التأمين لتغطية تكاليف الخدمة المقدمة؟)

1. **Confidentiality**

Q: (How do you maintain privacy and confidentiality when providing services?)

(كيف بتحافظو على الخصوصية والسرية عند تقديم الخدمات)

Does it include?

- private counseling spaces
- safe storage of files and non-disclosure of health information to parents or others without client permission

1. **Health Indicators**

Q: (How do you monitor and evaluate your services?)

(كيف تقيسون من فعالية/نجاح الخدمات المقدمة؟)

(كيف تتأكدون من فعالية/نجاح الخدمات المقدمة؟)

1. **Partnership/collaboration with other stakeholders**

Q: (هل يوجد شراكة أو تعاون مع جهات أخرى لتقديم الخدمات؟)

1. **Involvement of adolescents**

Q: (Do you involve adolescents at any stage in the services provision process?)

(هل يتم إشراك المراهقين/الشباب/اليافعين في أي مرحلة من مراحل عملية تقديم أو التخطيط للخدمات؟)

1. **Service Financing**

Q: (هل يغطي تكلفة هذه الخمة هذه الخدمه مصدر تمويل ذاتي أو خارجي)

1. **Other stakeholders**

Q: (To your knowledge can you provide us with adolescent services providers in Palestine?)

(ما هي المؤسسات الأخرى لبتي تعمل أو تقدم الخدمات للشباب / المراهقين / اليافعين؟)
